# Study on the spatial distribution characteristics and influencing factors of older adult(s) care facilities in Liaoning Province based on POI data

**DOI:** 10.3389/fpubh.2026.1735719

**Published:** 2026-02-10

**Authors:** Jiya Sun, Xiaojie Xue, Zheng Cui

**Affiliations:** 1 School of Management, Liaoning University of International Business and Economics, Dalian, China; 2 School of Marxism, Chang'an University, Xi'an, China; 3 School of Geographical Sciences, Liaoning Normal University, Dalian, China

**Keywords:** older adult(s) care facilities, geographic detector, influencing factors, Liaoning Province, spatial distribution

## Abstract

With the improvement of people's living standards and health conditions, enhancing the quality of life for the older adult(s) has become a pressing social issue that needs to be addressed. As an effective way to address China’s older adult(s) care challenges, the spatial layout of older adult(s) care facilities plays a crucial role in optimizing older adult(s) care resources and actively responding to population aging. Using spatial analysis methods and ArcGIS, this study analyzes the spatial density, spatial correlation, and spatial distribution patterns of older adult(s) care facilities in Liaoning Province, and employs geographical detectors to examine the factors influencing their spatial distribution. The results show that: ① In terms of spatial distribution, older adult(s) care facilities in Liaoning Province are more numerous in the central region and fewer in the surrounding areas. ② In terms of spatial clustering, older adult(s) care facilities have formed a high-density core area centered on Shenyang, a Bohai Rim clustering belt, and sub-core density areas centered on Dandong and Fuxin. ③ In terms of spatial matching between older adult(s) care facilities and the older adult(s) population, six cities have advanced older adult(s) care facility resources, four cities have basically matched resources, and four cities have lagging resources. ④ The factors influencing the distribution of older adult(s) care facility resources in Liaoning Province are complex, with the size of the older adult(s) population being the most important factor. These findings provide a scientific basis for optimizing the layout of older adult(s) care facilities in Liaoning Province, helping to rationally allocate older adult(s) care resources and promote the diversified development of older adult(s) care models

## 1 Introduction

With the improvement of people’s living standards and the advancement of medical technology, human life expectancy continues to increase. At present, China’s fertility rate is continuously declining, while the proportion of older adult(s) people in the population age structure is steadily rising. Therefore, population aging has become a basic national condition in China for the present and for a relatively long period in the future, and actively responding to population aging has been elevated to a national strategy (1), According to the “2024 National Report on the Development of Aging” released by China’s Ministry of Civil Affairs (2), by the end of 2024, China’s population aged 60 and above had reached 310.31 million, accounting for 22.0% of the total population, while the population aged 65 and above reached 220.23 million, accounting for 15.6%, indicating that China has entered a moderately aging society. Against the background of increasing population aging, exploring the spatial distribution of older adult(s) care facilities can not only address the older adult(s) care needs of local residents but also meet the development needs of emerging older adult(s) care models such as residential tourism for older adult(s) care (3).

Elderly care facilities refer to the general term for buildings that provide specialized or comprehensive services for the older adult(s) in terms of residence, daily care, medical care, cultural entertainment, and other aspects, including nursing homes, retirement homes, and older adult(s) day care centers (4, 5). Elderly care resources refer to the sum of elements that support the living, entertainment, and health needs of the older adult(s). Elderly care facilities are an important component of older adult(s) care resources and a key platform for their effective functioning (6). As a global research hotspot, studies on the quality of life of the older adult(s) population in Northeast China are conducive to providing Chinese solutions for the sustainable development of global older adult(s) care resources (7).

Some developed countries that entered an aging society earlier provide valuable references and experience for China in actively responding to population aging through research and practice on the distribution and rational planning of older adult(s) care resources. Foreign studies mainly focus on the residential preferences of the older adult(s) (8, 9), the demand for older adult(s) care facilities by the aging population (10–12), intelligent design of older adult(s) care facilities (13), evaluation standards of older adult(s) care service quality, and the construction of community-based older adult(s) care models (14–17). Nordic countries, especially Sweden, pay close attention to the physical and public health of the older adult(s) through research on community-based older adult(s) care (18). Norwegian scholars further explore older adult(s) mental health and social interaction through social robots (19). Scholars from Western Europe focus more on disease prevention in nursing institutions (20), while American scholars emphasize mental health care in older adult(s) care institutions (21). These studies indicate that increasing the quantity and improving the service quality of older adult(s) care institutions are important approaches to addressing older adult(s) care issues. This study focuses on analyzing the factors that lead to regional differences in the number of older adult(s) care facilities, providing references for the rational allocation of older adult(s) care resources in other countries and regions.

Research by Chinese scholars on older adult(s) care facilities mainly focuses on the following aspects. First, the spatial layout and site selection of older adult(s) care facilities (22), with research objects mainly concentrated in more developed cities (23, 24). By analyzing factors such as population aging and economic development levels (25, 26), optimization measures for facility layout and site selection are proposed (27, 28). Second, accessibility analysis of older adult(s) care facilities based on multi-source data such as population, transportation, and facility distribution (29, 30). Methods including the two-step floating catchment area method, grid cost-distance algorithm, and coverage method are widely used to measure accessibility, helping to improve service systems and resource utilization efficiency (31, 32). Third, research on the matching between older adult(s) care facilities and population aging examines whether supply and demand are balanced and provides planning suggestions based on aging trends (33). Fourth, studies on intelligent older adult(s) care facility design promote smart older adult(s) care through big data and intelligent technologies, improving service quality and management models (34, 35). Fifth, research on spatial equity of older adult(s) care facilities shows that significant spatial clustering exists, and vulnerable groups such as rural left-behind older adult(s), older adult(s) who have lost their only child, and older adult(s) living alone are often overlooked (36–38). In addition, government policies play an important role in facility planning, as the household registration system and urban–rural differences in social security restrict the development of older adult(s) care facilities (39, 40). Numerous studies based on yearbook data, older adult(s) care industry statistics, and livelihood data demonstrate that rational planning of older adult(s) care facilities is crucial for improving the quality of life in China. This study uses consistency coefficients to infer the rational layout of older adult(s) care facilities in Liaoning Province, contributing to better protection and development of older adult(s) care resources and offering insights for addressing global older adult(s) care challenges.

Liaoning Province has a large and continuously growing older adult(s) population and has entered a severely aging society. According to the “2023 Liaoning Provincial Report on the Development of Aging Affairs” (41), by the end of 2023, the province’s older adult(s) population aged 60 and above had reached 12.3 million, accounting for 29.4% of the total population, while the population aged 65 and above reached 8.81 million, accounting for 21.1%. The degree of aging ranks first nationwide, with the older adult(s) dependency ratio (aged 65 and above) reaching 30.6%. Existing studies on older adult(s) care in Liaoning Province mainly focus on the construction of older adult(s) care service systems (42), service quality of institutional care (43), demand for older adult(s) care real estate (44), and rural older adult(s) care issues (45). However, despite being the most severely aging region in China, in-depth research on the spatial distribution characteristics of older adult(s) care facilities in Liaoning Province remains limited.

In this context, this paper uses POI data of older adult(s) care facilities in Liaoning Province in 2024 and applies ArcGIS to analyze their spatial distribution characteristics and matching degree across cities. Factor detection and interaction detection based on the geographical detector method are employed to identify the influencing factors of spatial distribution. The specific objectives are as follows: (1) to comprehensively present the spatial distribution of older adult(s) care facilities in Liaoning Province through methods such as the nearest neighbor index and kernel density analysis; (2) to analyze the matching degree of older adult(s) care facilities among cities using consistency coefficients, thereby enriching related research; and (3) to identify key influencing factors through geographical detector analysis and propose differentiated policy suggestions. This study provides practical references for the efficient utilization of older adult(s) care resources, the diversified development of older adult(s) care models, and the rational planning of urban development.

## 2 Data sources and methods

### 2.1 Research framework

**Figure 1:**
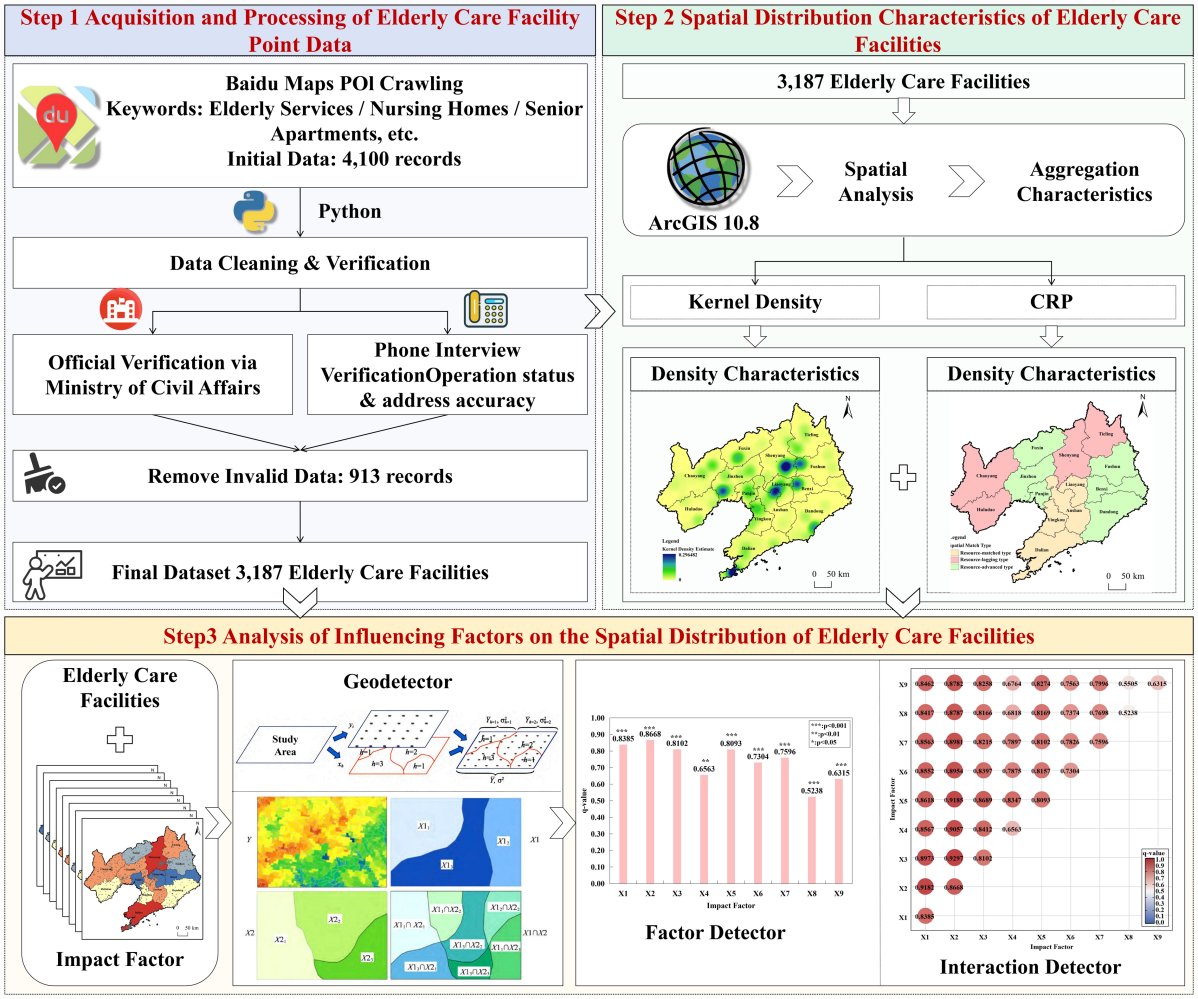
Research framework diagram. Map drawn using ArcGIS software, adapted from the China Surveying, Mapping and Geographic Information Standard Map Service website (http://bzdt.ch.mnr.gov.cn/), Map No. GS (2019) 1823, Map Technology Review Center, Ministry of Natural Resources, China.

This study establishes a research framework for analyzing the spatial distribution of older adult(s) care facilities in Liaoning Province and their multi-scale influencing factors (Figure 1). The framework divides the research process into three steps: Step 1 focuses on acquiring and processing point data on older adult(s) care facilities; Step 2 describes the spatial distribution characteristics of these facilities; Step 3 analyzes the factors influencing their spatial distribution.

### 2.2 Data sources

Liaoning Province is located in Northeast China, covering an area of 148,000 square kilometers. The province has abundant older adult(s) care resources and is nationally renowned for its hot spring–based, pristine natural environment–based, and coastal older adult(s) care services. Liaoning ranks first nationwide in terms of population aging and has a large number of older adult(s) care facilities; therefore, it was selected as the study area. This study employed web crawling technology in combination with the China Elderly Care Information Service Platform^1^, utilizing defined POI queries. Using “older adult(s) services” as the primary keyword, “nursing homes” as the secondary keyword, and “senior apartments,” “older adult(s) care,” and “senior activity centers” as tertiary keywords, POI data on older adult(s) care facilities in Liaoning’s 14 prefecture-level cities in 2024 were extracted from Baidu Maps, yielding 4,100 sample records. During the sample verification stage, the operational status of these facilities was cross-checked using the China Elderly Care Institutions Database of the Ministry of Civil Affairs^2^. Telephone interviews were conducted to confirm operational status and address accuracy. As a result, 913 facilities were removed due to duplication, closure, or abnormal operation, leaving 3,187 older adult(s) care institutions in Liaoning Province. This dataset forms the basis for analyzing the spatial distribution characteristics of older adult(s) care facilities.

**Figure 2:**
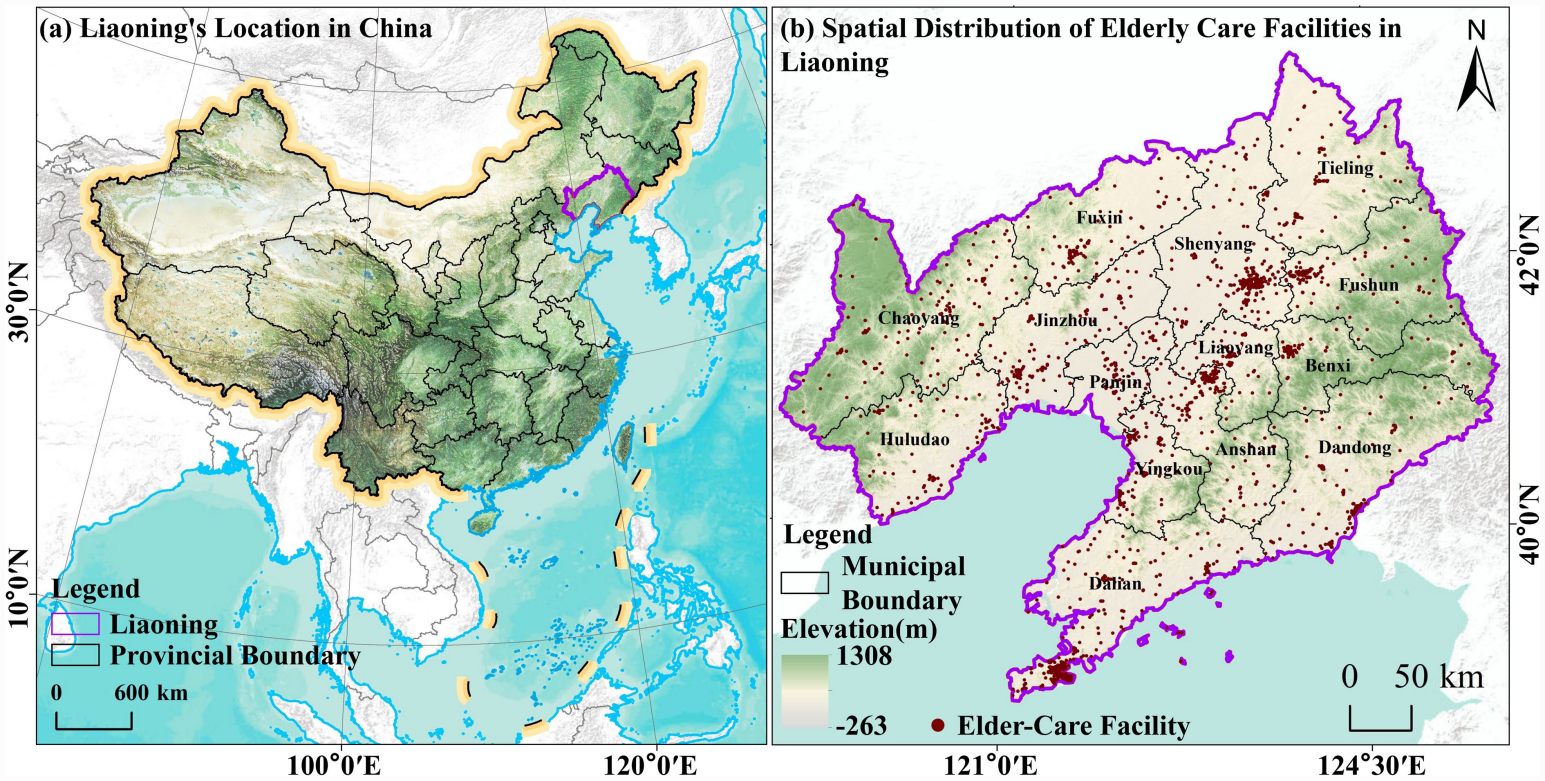
Spatial distribution map of elderly care facilities in Liaoning Province. Maps created with ArcGIS software (https://www.arcgis.com/index.html).

**Figure 3:**
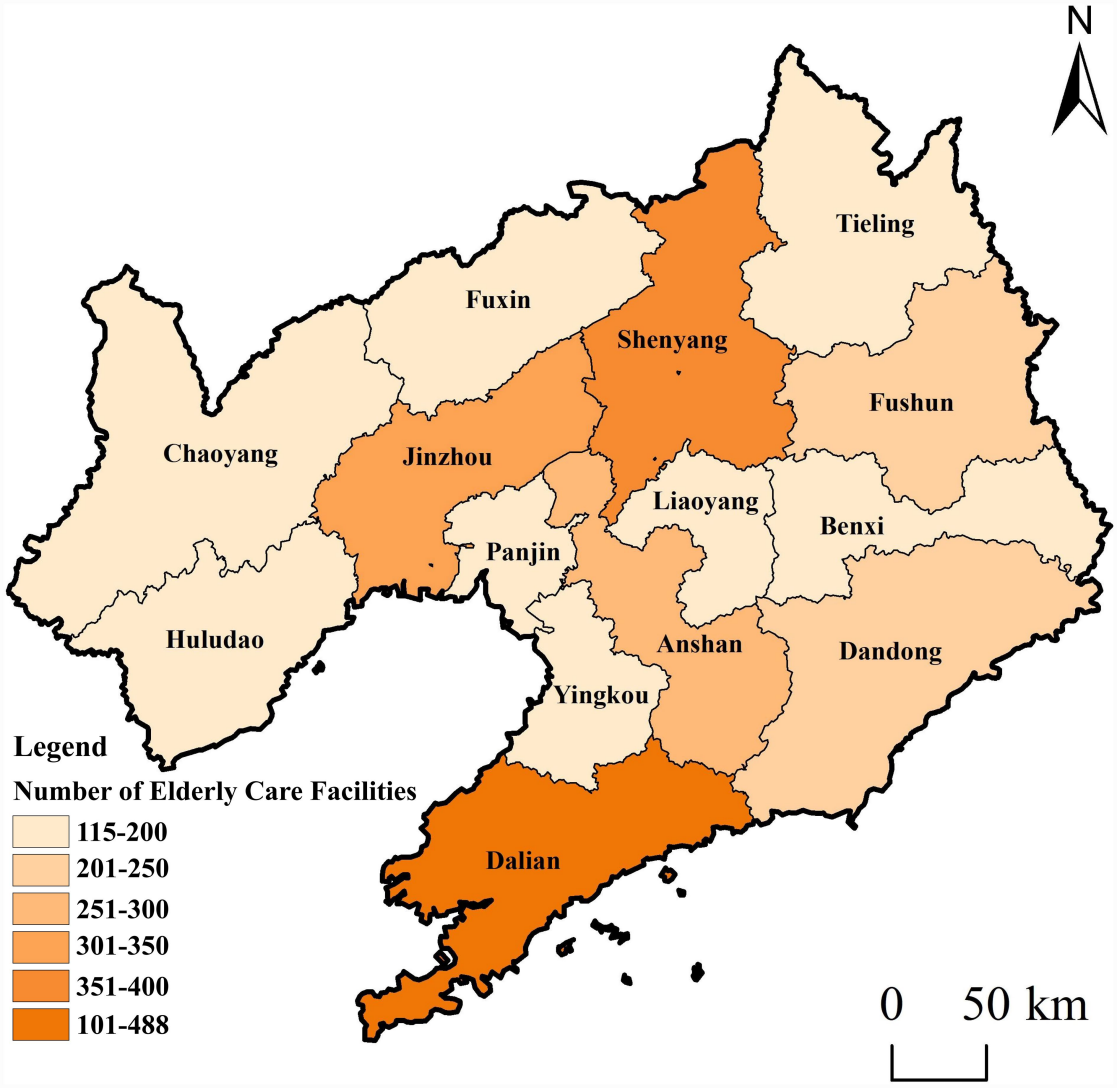
Distribution map of the number of elderly care facilities by City in Liaoning province. Maps created with ArcGIS software (https://www.arcgis.com/index.html).

Using map coordinate pickers, geographic coordinates were obtained based on the location information of older adult(s) care facilities. At the provincial scale, the data were treated as point features. ArcGIS software was used to perform coordinate and projection transformations, generating a spatial distribution map of older adult(s) care facilities in Liaoning Province (Figure 2). At the same time, based on the number of older adult(s) care facilities in each of Liaoning’s 14 prefecture-level cities, the point data were aggregated into polygon data to produce a map showing the distribution of older adult(s) care facilities by city (Figure 3).

### 2.3 Selection of influencing factors

To analyze the spatial distribution characteristics and influencing factors of older adult(s) care facilities across Liaoning cities, six categories of influencing factors were selected from economic, social, and environmental perspectives. These include population size, economic development level, tourism resources, healthcare standards, transportation accessibility, and environmental quality, represented by nine indicators: total population (X1), older adult(s) population (X2), urban GDP (X3), number of tourist attractions (X4), number of medical facilities (X5), transportation accessibility (X6), average January temperature (X7), average August temperature (X8), and air quality (X9).

The influencing factors are explained as follows (Table 1). ① Population size includes total population and older adult(s) population. A larger older adult(s) population increases demand for older adult(s) care facilities, while population base and demographic structure determine aging trends and future demand; therefore, these indicators are expected to be strongly correlated with facility distribution. ② Economic development level, measured by urban GDP, reflects a city’s capacity to supply older adult(s) care services. Higher GDP indicates stronger investment capability and is expected to correlate with the quantity and spatial distribution of older adult(s) care facilities. ③ Tourism resources are represented by the number of tourist attractions. Web crawling was used to collect POI data on A-level scenic sites, parks, museums, and recreational plazas. Rich tourism resources indicate a high-quality living environment and are expected to attract older adult(s) residents and stimulate demand for older adult(s) care services. ④ Medical standards are measured by the number of medical facilities, including general hospitals, specialized hospitals, and community clinics. Convenient access to healthcare is essential for older adult(s) daily life, and cities with higher medical standards are expected to host more older adult(s) care facilities. ⑤ Transportation convenience is measured by road network density in urban built-up areas. Cities with better transportation accessibility are expected to support a wider distribution of older adult(s) care facilities. ⑥ Environmental quality is reflected by average January temperature, average August temperature, and air quality. Elderly populations generally prefer locations with mild winters, cool summers, and good air quality; thus, environmental quality is an important indicator of urban livability and is expected to influence the spatial distribution of older adult(s) care facilities.

**Table 1:** Data sources of influencing factor indicators for elderly care facilities in Liaoning Province.

Influencing factors	Indicator variables	Data source	Remarks
Population size	Total population (X1)	From each City Statistics Bureau in Liaoning Province	Permanent resident population
	elderly population (X2)		Aged 60 and above
The level of economic development	Urban GDP (X3)	Communiqué on National Economic and Social Development of each city in Liaoning Province	
Tourism resources	Number of tourist attractions (X4)	Baidu Map POI Acquisition	Grade A tourist attractions, parks, museums, leisure squares, etc.
The level of medical care	Number of healthcare facilities (X5)	Baidu Map POI Acquisition	Various medical service institutions
Traffic convenience	Traffic accessibility (X6)	National Fundamental Geographic Information System	
Environmental quality	Average temperature in January (X7)	Liaoning Provincial Meteorological Bureau	
	Average temperature in August (X8)		
	Air quality (X9)	Liaoning Provincial Department of Ecology and Environment	

### 2.4 Research methods

#### 2.4.1 Nearest neighbor index

The nearest neighbor index refers to the proximity distance between a point and its nearest feature point within a specific area. The magnitude of the nearest neighbor index can be used to measure the degree of clustering in the distribution of point features (46). The calculation formulas are as follows:


$$R=\frac{{\bar{\gamma }}_{a}}{{\bar{r}}_{E}}$$ (1)

$${\bar{r}}_{E}=\frac{1}{2\sqrt{\frac{n}{A}}}=\frac{1}{2\sqrt{D}}$$ (2)

In the formula: ${\bar{\gamma }}_{a}$ is the average distance between all older adult(s) care facility points within the study area and their nearest spatial points, ${\bar{r}}_{E}$ representing the theoretical nearest neighbor distance when older adult(s) care facilities are randomly distributed; n is the number of older adult(s) care facilities within the study area; A is the total area of the entire study region; R is the nearest neighbor index. When R < 1, R = 1, or R > 1, it indicates that older adult(s) care facilities tend to be clustered, uniformly, or randomly distributed, respectively. Moreover, the smaller the R value, the greater the degree of clustering.

#### 2.4.2 Kernel density analysis

Kernel Density Analysis uses the Kernel Density tool in ArcGIS Spatial Analyst. Based on the location information of older adult(s) care facilities in Liaoning Province obtained through web crawling, this method calculates the density of points around each output raster cell, providing a more intuitive representation of the distribution density of older adult(s) care facilities and their degree of spatial clustering or dispersion (47). The formula is:


$$f_{\left(x\right)}=\frac{1}{\mathrm{nh}}\sum_{i=1}^{n}k(\frac{x-x_{i}}{h})$$ (3)

In the formula: h represents the bandwidth; n is the total number of older adult(s) care facilities; x-xi denotes the distance from older adult(s) care facility x to x_i_.

#### 2.4.3 Consensus rate percentage

The Consistency Ratio Percentage (CRP), also referred to as the matching degree, is calculated as the ratio between the proportion of older adult(s) care facilities in each city of Liaoning Province and the proportion of that city’s older adult(s) population in the total older adult(s) population of the province. This index reflects the degree of consistency between older adult(s) care facilities and the older adult(s) population in each city (47). The formula is as follows:


$$\mathrm{CR}P_{i}=\frac{R_{\mathrm{re}s_{i}}}{P_{\mathrm{po}p_{i}}}=\frac{\mathrm{re}s_{i}/\sum \mathrm{re}s_{i}}{\mathrm{te}r_{i}/\sum \mathrm{te}r_{i}}∕\frac{\mathrm{po}p_{i}/\sum \mathrm{po}p_{i}}{\mathrm{te}r_{i}/\sum \mathrm{te}r_{i}}=\frac{\mathrm{re}s_{i}/\sum \mathrm{re}s_{i}}{\mathrm{po}p_{i}/\sum \mathrm{po}p_{i}}$$ (4)

In the formula: pop_i_ and res_i_ represent the older adult(s) population and total older adult(s) care resources in region i during a certain period, respectively; ter_i_ represents the area of region i; ∑pop _i_、 ∑res _i_ and ∑ter _i_ represent the total older adult(s) population, total older adult(s) care facilities, and total geographical area of the 14 prefecture-level cities in Liaoning Province, respectively. In the calculation results, the larger the CRP value, the higher the agglomeration degree of older adult(s) care facilities in the region. Among them, CRP>1 indicates that the agglomeration degree of older adult(s) care facilities in the region has exceeded the concentration of older adult(s) population distribution, belonging to the resource-advanced type; CRP = 1 indicates that the agglomeration degree of older adult(s) care facilities and older adult(s) population distribution in the region is consistent, belonging to the resource-matching type; CRP<1 indicates that the agglomeration level of older adult(s) care facilities in the region is lower than the concentration of older adult(s) population distribution, belonging to the resource-lagged type.

#### 2.4.4 Geodetector

Geodetector is a tool used to detect and analyze spatial heterogeneity. Through factor detection and interaction detection, it examines the relationship between the dependent variable (number of older adult(s) care facilities) and independent variables (influencing factors X1–X9), and measures the explanatory power of the independent variables on the dependent variable using the q-value (48). The formula is:


$$q=P_{X,Y}=1-\frac{1}{n\sigma^{2}}\sum_{i=1}^{m}n_{i}\sigma_{i}^{2}$$ (5)

In the formula: Y is the number of older adult(s) care facilities, P_X, Y_ is the influence of independent variable X on dependent variable Y; n is the number of samples; $\sigma^{2}$ is the variance of all regions, m is the number of strata of Y, and $\sigma_{i}^{2}$ is the variance of sub-regions. q ranges between 0 and 1, with a higher q-value indicating a stronger influence of independent variable X on dependent variable Y.

## 3 Results and analysis

### 3.1 Spatial distribution characteristics of older adult(s) care facilities

#### 3.1.1 Spatial cluster characteristics of older adult(s) care facilities

At the municipal scale, taking the 14 prefecture-level cities of Liaoning Province as the study units, the nearest neighbor index was calculated using Equations 1, 2 (Table 2). The nearest neighbor index (R) values for all 14 cities are below 0.5 and have passed the significance test, indicating that the spatial distribution of older adult(s) care facilities in Liaoning Province exhibits a clustered pattern. Among them, Benxi City has the lowest nearest neighbor index (0.280), indicating the highest degree of clustering.

**Table 2:** Nearest neighbor index and spatial distribution types of elderly care facilities in each city of Liaoning Province.

City	Actual average nearest neighbor (km)	Theoretical average nearest neighbor (km)	*P*-value	*Z*-score	Nearest neighbor index R	Spatial distribution type
Benxi	0.97	3.46	0	−18.886	0.280	Cluster distribution
Dandong	1.54	4.49	0	−19.573	0.342	Cluster distribution
Tieling	1.82	4.97	0	−15.074	0.367	Cluster distribution
Jinzhou	1.10	2.97	0	−21.505	0.370	Cluster distribution
Dalian	1.39	3.71	0	−26.434	0.375	Cluster distribution
Panjin	0.87	2.25	0	−12.596	0.386	Cluster distribution
Shenyang	1.30	3.12	0	−21.924	0.416	Cluster distribution
Yingkou	1.19	2.80	0	−14.976	0.426	Cluster distribution
Fushun	1.78	3.82	0	−15.136	0.467	Cluster distribution
Anshan	1.52	3.18	0	−16.765	0.479	Cluster distribution
Liaoyang	1.55	3.00	0	−10.871	0.518	Cluster distribution
Chaoyang	3.65	6.03	0	−10.544	0.607	Cluster distribution
Fuxin	2.48	4.01	0	−9.005	0.618	Cluster distribution
Huludao	3.11	4.79	0	−7.307	0.650	Cluster distribution
Liaoning Province	1.58	4.29	0	−28.117	0.369	Cluster distribution

#### 3.1.2 Spatial distribution density characteristics of older adult(s) care facilities

**Figure 4:**
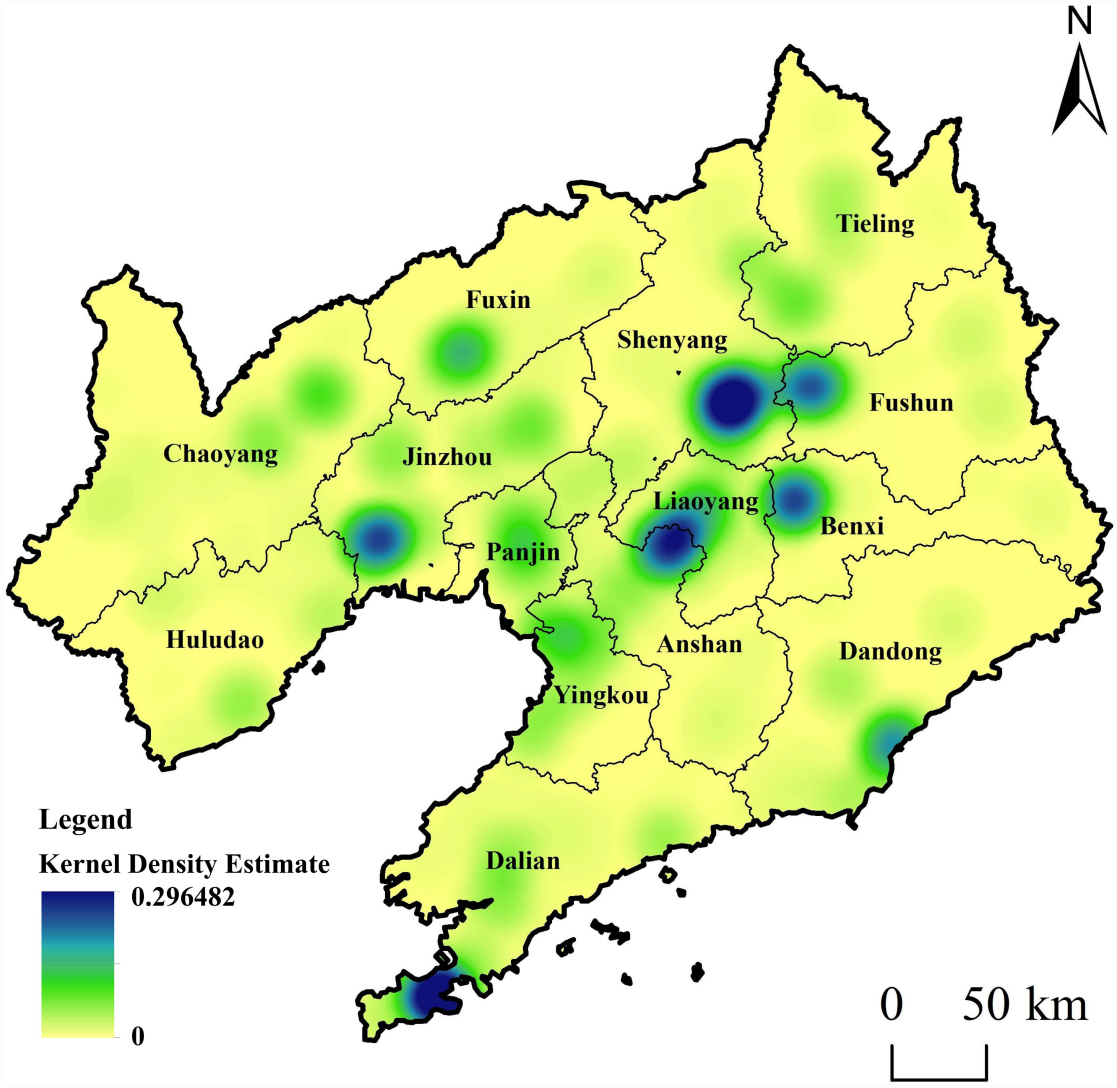
Spatial distribution map of kernel density of elderly care facilities in Liaoning Province. Maps created with ArcGIS software (https://www.arcgis.com/index.html).

This study adopts the kernel density analysis method (Equation 3), and determines the optimal bandwidth as 15.2 kilometers (search radius) based on Silverman’s empirical rule. The unit of kernel density value is number of facilities per square kilometer (facilities/km^2^), with a value range from 0 to 0.296482, reflecting the concentration degree of older adult(s) care facilities per unit area. Based on the POI data of older adult(s) care facilities in Liaoning Province, a kernel density spatial distribution map of older adult(s) care facilities in Liaoning Province was generated (Figure 4). The spatial distribution of older adult(s) care facilities in Liaoning Province is uneven at the provincial scale and presents a pattern of “overall dispersion with sporadic aggregation.” At the provincial level, a high-density core centered on Shenyang and a Bohai Rim clustering belt connecting Jinzhou, Panjin, Yingkou, and Dalian have formed. In addition, Dandong and Fuxin have developed two smaller secondary kernel density clusters.

Shenyang and Dalian are the two core cities of economic and social development in Liaoning Province. These cities occupy leading positions in terms of population size, economic development, and transportation accessibility, and their older adult(s) care facilities form high-density core areas. The Bohai Rim cluster belt of older adult(s) care facilities connecting Jinzhou, Panjin, Yingkou, and Dalian is consistent with the Liaoning Coastal Economic Belt, which is a key development zone in the province. The level of urban development and economic strength in this coastal belt is among the highest in Liaoning Province. At the same time, the region enjoys favorable environmental and climatic conditions, contributing to a dense distribution of older adult(s) care facilities. In contrast, cities such as Tieling, Chaoyang, and Huludao exhibit lower levels of older adult(s) care facility clustering, which is closely related to factors such as population size, economic development level, and medical standards. These cities have relatively low per capita GDP and smaller populations within the province, resulting in weaker spatial clustering of older adult(s) care facilities.

### 3.2 Analysis of matching types between older adult(s) care facilities and aging population

This study sets the range of 0.9–1.1 as the discriminant threshold for “resource-matching type.” This threshold setting is based on the practical consideration that theoretical equilibrium (CRP = 1) is difficult to achieve in actual resource configuration. Referencing the commonly used ±10% fluctuation range in supply–demand matching research, this reflects the reasonable elasticity range allowed between the supply and demand of older adult(s) care services. To verify the robustness of this threshold, this study conducted a sensitivity analysis, reclassifying urban types using 0.85–1.15 (lenient threshold) and 0.95–1.05 (strict threshold) respectively.

The results of the sensitivity analysis are shown in Table 3: compared to the baseline threshold (0.9–1.1), the relaxed threshold only shifted one city from the matching type to the resource-advanced type. Strict thresholds shift two cities from the matching type to resource-lagging cities, with overall classification consistency rates of 92.9 and 85.7%, respectively. The results indicate that the threshold selected in this paper exhibits good robustness within a reasonable fluctuation range.

**Table 3:** CRP threshold robustness test results.

Threshold setting	Number of resource-advanced cities	Number of resource-matched cities	Number of resource-lagged cities	Comparison with baseline threshold
Baseline threshold (0.9–1.1)	6	4	4	100%
Loose threshold (0.85–1.15)	7	3	4	92.9%
Strict threshold (0.95–1.05)	6	2	6	85.7%

**Figure 5:**
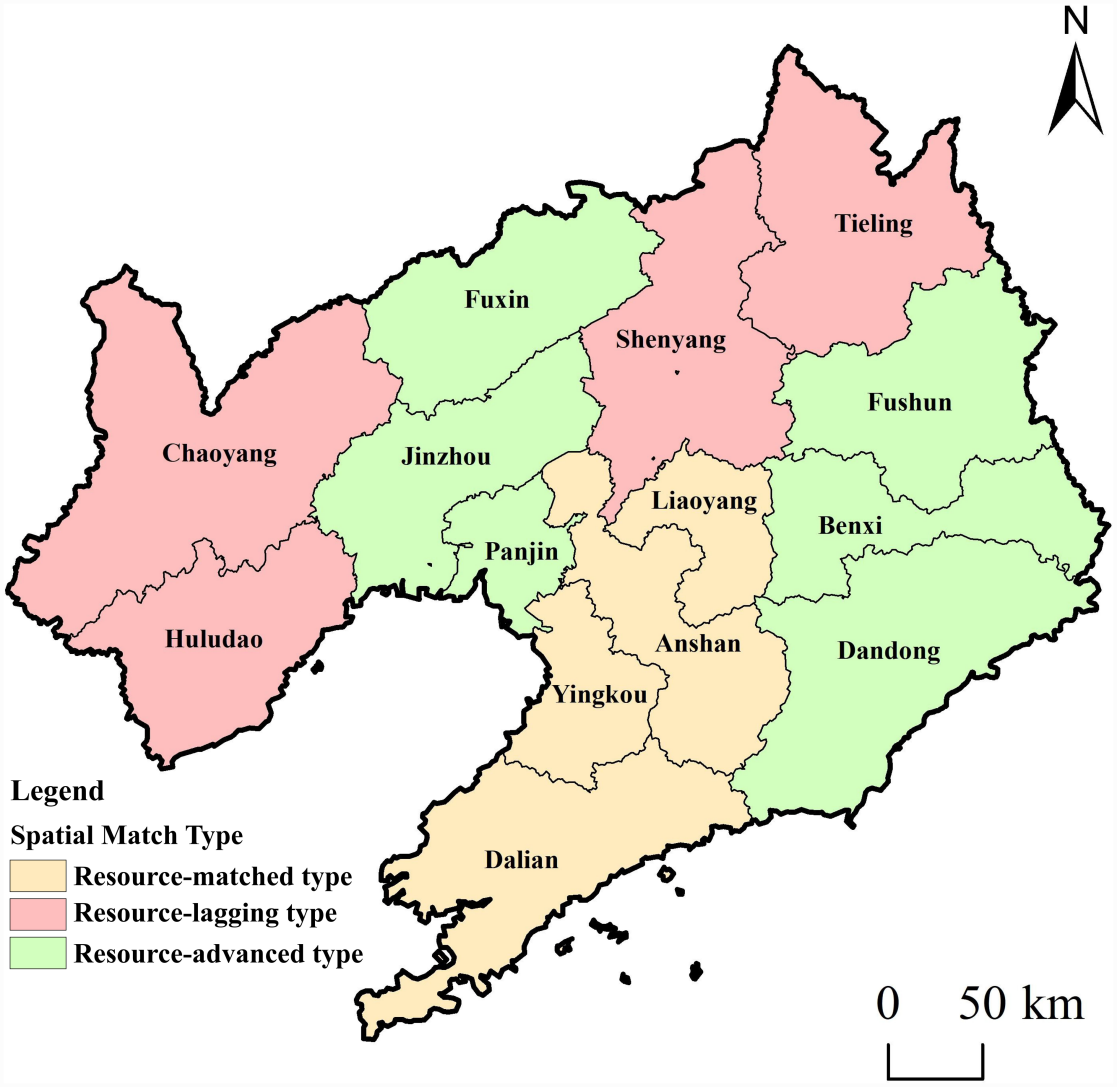
Spatial matching analysis of elderly care facilities in each city in Liaoning Province. Maps created with ArcGIS software (https://www.arcgis.com/index.html).

Based on the number of older adult(s) care facilities and the older adult(s) population in each city (Equation 4), the consistency coefficient between older adult(s) care facilities and the older adult(s) population at the municipal scale was calculated to analyze the matching status of older adult(s) care facilities across Liaoning Province (Figure 5). To clearly classify matching types, cities with a higher concentration of older adult(s) care facilities than older adult(s) population (CRP > 1.1) are defined as resource-advanced; cities with roughly equivalent concentrations (0.9 ≤ CRP ≤ 1.1) are defined as resource-matching; and cities with a lower concentration of older adult(s) care facilities than older adult(s) population (CRP < 0.9) are defined as resource-lagging, indicating insufficient provision and a need for increased investment.

According to the matching situation of older adult(s) care facilities and older adult(s) population in cities of Liaoning Province (Table 4), there are 6 cities in Liaoning Province where the construction of older adult(s) care facilities and older adult(s) population distribution show a “resource-advanced type,” namely Benxi, Jinzhou, Dandong, Panjin, Fushun and Fuxin, accounting for 42.86% of the province. The economic development levels of these cities are all in the middle or lower ranks in the province, with smaller populations. Except for Jinzhou and Dandong with populations exceeding 2 million, the resident populations of the other 4 cities are all below 2 million. Consequently, the overall development level of older adult(s) care facilities in resource-advanced cities is not high; rather, their classification as resource-advanced mainly results from smaller total and older adult(s) populations, leading to a relatively high per capita allocation of facilities.

**Table 4:** Spatial matching types of elderly care facilities and elderly population in cities of Liaoning Province.

City	Elderly population (approximate value)	Elderly care facilities	Consensus rate percentage (CRP)	Matching types
Benxi	470,000	188	1.544	Resource-advanced type
Jinzhou	879,000	318	1.396	Resource-advanced type
Dandong	716,000	242	1.304	Resource-advanced type
Panjin	345,000	115	1.286	Resource-advanced type
Fushun	683,600	220	1.242	Resource-advanced type
Fuxin	519,000	152	1.130	Resource-advanced type
Yingkou	646,000	186	1.099	Resource-matched type
Anshan	1,011,000	283	1.080	Resource-matched type
Dalian	1,826,900	488	1.031	Resource-matched type
Liaoyang	528,000	139	1.016	Resource-matched type
Chaoyang	862,000	197	0.882	Resource-lagging type
Tieling	799,000	155	0.749	Resource-lagging type
Shenyang	2,240,000	385	0.663	Resource-lagging type
Huludao	774,500	119	0.593	Resource-lagging type

Four cities—Yingkou, Anshan, Dalian, and Liaoyang—belong to the resource-matching category, accounting for 28.57% of the total. Liaoyang, Anshan, and Yingkou are all located in central–southern Liaoning (Figure 5), where close socioeconomic linkages have formed a pattern of regional resource sharing and functional complementarity. As traditional industrial bases, Anshan and Liaoyang, together with Yingkou as a port city, have long-standing interactions in industrial development and population mobility, providing a foundation for coordinated allocation of older adult(s) care resources. In addition, high transportation accessibility among these cities facilitates cross-city use of older adult(s) care services and encourages institutions to consider regional service coverage when planning facility layouts. Dalian benefits from unique natural endowments, including a favorable climate, an extensive coastline, and abundant leisure and older adult(s)-friendly spaces such as tourist attractions, parks, and green areas. It also possesses strong comprehensive economic strength, complete infrastructure, and a relatively mature social security system. As a result, despite having a large older adult(s) population, Dalian achieves a basic balance between older adult(s) care facilities and demand. Moreover, through a combined “siphon–radiation” effect, Dalian attracts older adult(s) residents from surrounding areas seeking a higher quality of life, while its transportation advantages reduce both psychological and physical barriers to cross-regional older adult(s) care.

There are four cities in which the construction of older adult(s) care facilities and the distribution of the older adult(s) population exhibit a resource-lagging pattern: Chaoyang, Tieling, Shenyang, and Huludao. The lag in older adult(s) care resources in these areas results from multiple factors, including economic structure, population mobility, fiscal capacity, and geographical location. First, cities such as Chaoyang and Tieling have experienced slowing economic growth due to resource depletion and industrial decline, leading to insufficient financial support for older adult(s) care resources. As an old industrial base, Huludao has a relatively single industrial structure, and limited local fiscal revenue has constrained investment in public services such as older adult(s) care. Although Shenyang, as the provincial capital, leads the province in the total number of older adult(s) care facilities, it faces a pronounced “center-periphery” imbalance: high-quality older adult(s) care resources are concentrated in central urban areas, while suburban areas and older communities lack adequate provision. In addition, as the provincial capital, Shenyang broader development responsibilities, with a large share of investment directed toward economic sectors. As a result, despite some financial input into older adult(s) care, per capita resource coverage remains insufficient. Second, the aging rates in cities such as Chaoyang and Tieling have long ranked among the highest in Liaoning Province, causing older adult(s) care demand to exceed local resource carrying capacity. Shenyang also has a large older adult(s) population base; in 2024, its registered older adult(s) population exceeded two million, and the growth of older adult(s) care demand has outpaced the expansion of facilities. Finally, the lack of economic vitality in cities such as Chaoyang and Tieling has led to significant outmigration of young and middle-aged labor to Shenyang, Dalian, and other regions, weakening local purchasing power for older adult(s) care services and reducing the labor supply for the older adult(s) care industry.

## 4 Analysis of impact factors on the spatial distribution of older adult(s) care facilities

### 4.1 Analysis of factor detection results

**Figure 6:**
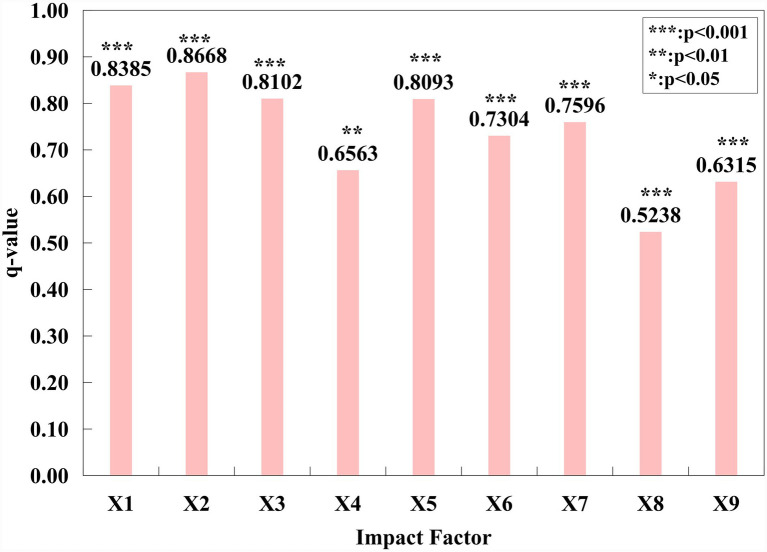
Analysis of the detection results of impact factors for elderly care facilities in Liaoning Province. ** Indicates significance at the 1% confidence level, * indicates significance at the 10% confidence level.

In this study, the number of older adult(s) care facilities in each city of Liaoning Province is taken as the dependent variable, while total population (X1), older adult(s) population (X2), urban GDP (X3), number of tourist attractions (X4), number of healthcare facilities (X5), transportation accessibility (X6), average January temperature (X7), average August temperature (X8), and air quality (X9) are used as independent variables. City-level explanatory variables were input into the geographical detector model, and R-language programs were used to select the optimal discretization method (Equation 5). The resulting *q*-values represent the influence of each indicator on the spatial distribution of older adult(s) care facilities across the 14 cities of Liaoning Province (Figure 6).

To enhance the model’s interpretability and the robustness of the results, before conducting formal analysis, this study systematically optimized the discretization processing of continuous independent variables using R language programs. The specific optimization process is as follows: the system compares four common discretization methods: natural breakpoint method, quantile method, geometric interval method, and standard deviation method, and sets each method with 3 to 10 categories for parameter debugging. The discretization scheme that maximizes the q-value of the geographical detector through cross-validation is selected as the final parameter for each variable. This optimization process aims to preserve the core features of the original data structure and maximize the explanatory power of each variable for the spatial distribution phenomenon of older adult(s) care facilities. The optimal discretization method for each variable and the final number of classifications are detailed in Table 5.

**Table 5:** Geographical detector variable optimal discretization scheme and classification number.

Variable	Optimal discretization method	Number of categories
X1	Natural breaks method	4
X2	Natural breaks method	5
X3	Natural breaks method	4
X4	Natural breaks method	4
X5	Natural breaks method	5
X6	Standard deviation method	5
X7	Quantile method	4
X8	Geometric interval method	3
X9	Quantile method	3

The q-value indicates the explanatory power of an independent variable X_i_ on the dependent variable Y, while the *p*-value reflects the statistical significance of the results. All *p*-values are less than 0.01, indicating that the results pass the significance test. The influence of factors, ranked by *q*-value, is as follows: older adult(s) population > total population > urban GDP > number of healthcare facilities > average January temperature > transportation accessibility > number of tourist attractions > air quality index > average August temperature. Among these, older adult(s) population, total population, urban GDP, and number of healthcare facilities all have q-values greater than 0.8, indicating a very strong influence on the spatial distribution of urban older adult(s) care facilities.

**Figure 7:**
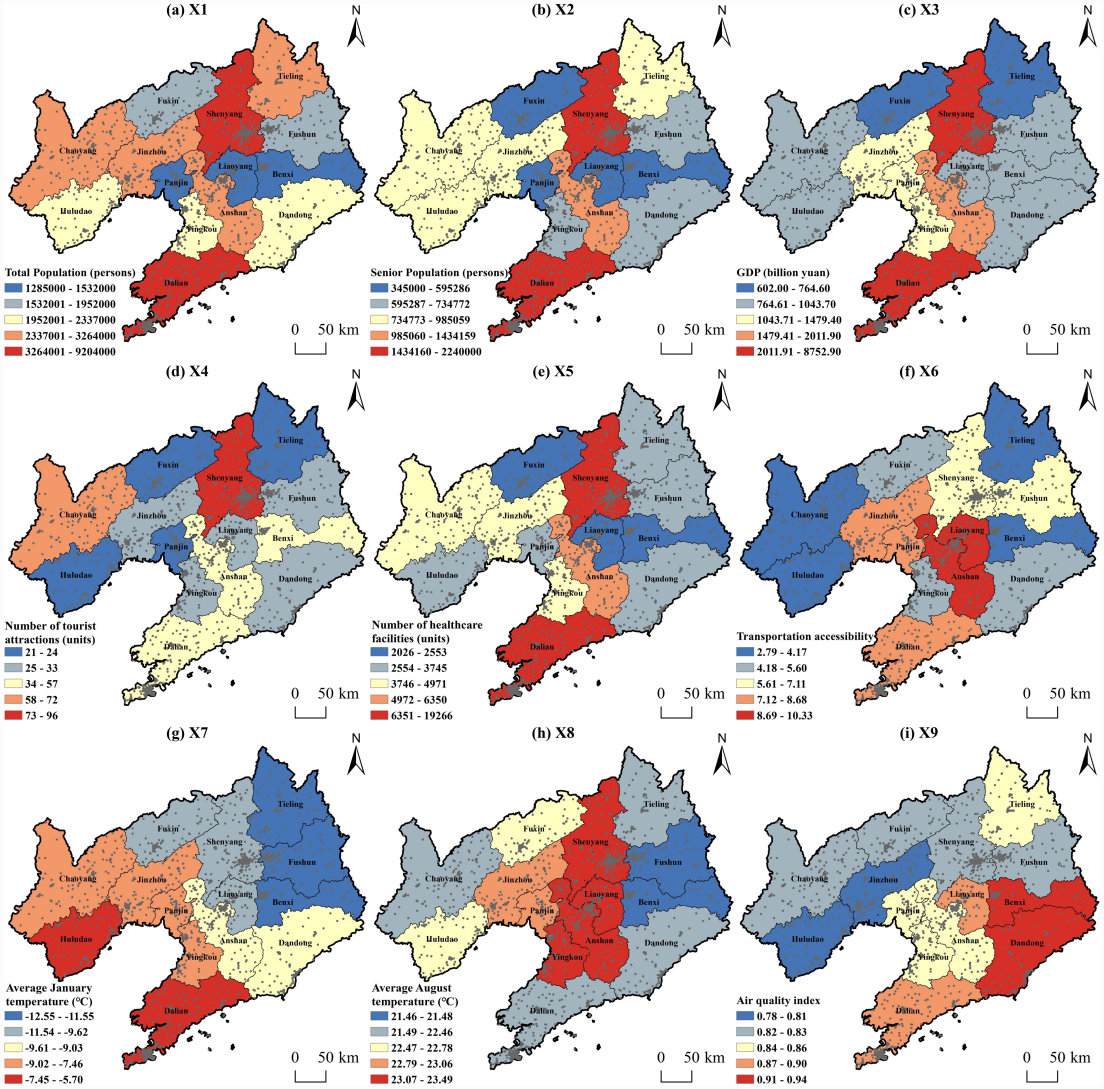
Analysis of impact factor indicators for elderly care facilities in Liaoning Province. Maps created with ArcGIS software (https://www.arcgis.com/index.html).

Based on the ranking of influencing factors and the spatial distribution characteristics of older adult(s) care facilities in Liaoning Province (Figure 7), The analysis of influencing factor indicators adopts the natural breaks method to uniformly classify all factors into five levels, which can determine the optimal classification breakpoints based on the distribution characteristics of the data. A detailed analysis of each indicator was conducted. Among all factors, the older adult(s) population exerts the greatest influence on the spatial distribution of older adult(s) care facilities. As a type of public resource, older adult(s) care facilities should adhere to a “demand-oriented” principle. The older adult(s) population constitutes the direct service group, and the number of older adult(s) residents in a region forms the fundamental basis for planning service staff, bed capacity, and capital investment (49).

Total population plays a foundational role in the allocation of older adult(s) care resources. While the older adult(s) population determines direct market demand, the total population serves as the starting point for overall infrastructure planning and directly affects infrastructure provision. It also reflects a city’s development potential and the economic feasibility of facility construction, thereby influencing the spatial distribution of older adult(s) care facilities (50).

Cities with higher levels of economic development generally have larger older adult(s) populations and more diversified demands. In such regions, both market forces and government capacity support increased supply and higher-quality facilities. In contrast, cities with lower economic development levels often face problems such as insufficient numbers of older adult(s) care facilities, low facility density, and inadequate service quality (50).

Elderly people are the group that uses medical services most frequently. Many older adult(s) individuals suffer from one or more chronic diseases, such as hypertension, diabetes, and cardiovascular or cerebrovascular conditions, which creates a strong dependence on medical resources. Therefore, the availability of healthcare facilities is a crucial consideration when selecting locations for high-quality older adult(s) care facilities.

Transportation accessibility affects not only the time cost for older adult(s) people to access other social services, but also the cost for family members and service providers to reach older adult(s) care facilities. Accessibility depends not only on physical distance, but also on transportation modes, travel time, economic cost, and physical and psychological burden. On the one hand, older adult(s) people prefer facilities that are convenient for family visits; on the other hand, very old, disabled, or non-driving older adult(s) individuals rely heavily on public transportation. Good transportation accessibility is therefore a key factor in ensuring equitable access to older adult(s) care resources, promoting social participation, and reducing social isolation among the older adult(s) (51).

Environmental factors exert influence by affecting the physiological health, behavioral patterns, and living costs of the older adult(s). Older adults have poor tolerance to high and low temperatures, and many chronic conditions are sensitive to changes in temperature and humidity. Therefore, environmental factors have a significant impact on the site selection of older adult(s) care facilities. Winters in Northeast China are relatively cold, so the average temperature in January has a greater influence on the site selection of older adult(s) care facilities than in August. Healthy and active seniors with choices tend to prefer older adult(s) care facilities with good air quality. Good air quality directly manifests as better living comfort and happiness. The number of tourist attractions is closely related to quality of life and spiritual needs, as abundant tourism resources provide older adult(s) people with more leisure, social, sports, and cultural activities.

### 4.2 Analysis of interactive detection results

**Figure 8:**
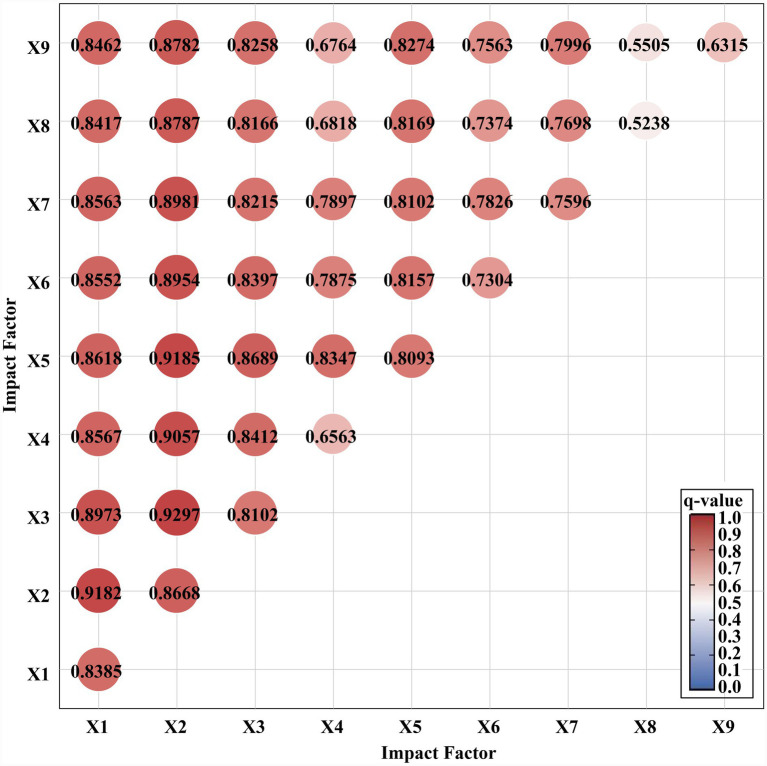
Interaction detection diagram of impact factors for elderly care facilities in Liaoning Province.

The spatial distribution characteristics of older adult(s) care facilities in Liaoning Province result from the combined effects of multiple factors, and their spatial patterns should therefore be examined comprehensively from multiple perspectives (Figure 8).

According to the interaction detection results of the geographical detector (Table 6), the explanatory power of all nine influencing factors is significantly enhanced after pairwise interaction, and all interactions belong to the bifactor enhancement type. This indicates that the spatial distribution of older adult(s) care facilities in Liaoning Province is shaped by the joint effects of multiple factors rather than by any single factor alone.

**Table 6:** Types of interaction in geographic detector.

Interaction type	Judgment basis
Non-linear weakening	q(X1∩X2) < min(q(X1),q(X2))
Single-factor non-linear weakening	min(q(X1),q(X2)) < q(X1∩X2) < max(q(X1),q(X2))
Dual-factor enhancement	q(X1∩X2) > max(q(X1),q(X2))
Independent	q(X1∩X2) = q(X1) + q(X2)
Non-linear enhancement	q(X1∩X2) > q(X1) + q(X2)

The interaction between older adult(s) population and urban GDP is most significant (0.9297), indicating that economically developed regions with larger older adult(s) populations can better promote the construction of older adult(s) care facilities. After the superposition of older adult(s) population and healthcare facilities, the q value reaches 0.9185, suggesting that areas with dense older adult(s) populations and well-developed medical facilities are more likely to form older adult(s) care facility clusters. The interaction between older adult(s) population and tourist attractions (0.9057), between tourist attractions and healthcare facilities (0.8347), and between transportation accessibility and healthcare facilities (0.8157) are also prominent. Therefore, the interaction effects of factors such as older adult(s) population and healthcare facilities are all greater than their single-factor influence, indicating that these factors have synergistic or mediating effects when affecting older adult(s) care resources. That is, under the superposition of factors such as healthcare facilities, tourist attractions, and transportation accessibility in a region, the older adult(s) population will more significantly influence the formation, development, and distribution characteristics of older adult(s) care facilities in that area. Regions with suitable climates, larger older adult(s) populations, developed economies, and more medical facilities are more conducive to the clustering of older adult(s) care facilities.

## 5 Discussion

The spatial distribution of older adult(s) care facilities in Liaoning Province exhibits clear regional differentiation. From a city-level perspective, the main findings are as follows:

1. With regard to influencing factors, the formation of the high-density core area centered on Shenyang is primarily driven by the size of the older adult(s) population and the level of economic development. This conclusion is generally consistent with the findings of Zhong Yang et al. (47), who analyzed the influencing factors of the spatial distribution of older adult(s) care facilities in Hunan Province. The agglomeration of older adult(s) care resources along the Bohai Rim forms a coastal clustering belt, where the main driving factors include economic development level, number of healthcare facilities, and air quality. This is also consistent with the results of Pan Yicheng et al. (31, 49), who examined the matching degree of older adult(s) care facilities and the factors influencing resource agglomeration in the Yangtze River Delta urban agglomeration. Therefore, future development of older adult(s) care resources should prioritize areas with stronger economic foundations, better air quality, higher medical standards, and relatively large older adult(s) populations.
2. Building on the geodetector framework proposed by Wang Jinsong et al. (48), this study emphasizes multifactor interaction analysis rather than single-factor explanations. The results show that the synergistic effects of multiple factors—such as economic conditions, medical resources, and environmental quality—exert a much stronger influence on the spatial distribution of older adult(s) care facilities than any individual factor alone (52). While four core determinants emerge at the provincial scale—older adult(s) population size, total population size, number of healthcare facilities, and urban economic development—the dominant factors vary across cities with different levels of resource matching. For example, Liaoyang City ranks relatively low among prefecture-level cities in terms of total population, healthcare resources, and economic development, yet it exhibits a high degree of resource matching due to its relatively small older adult(s) population. In contrast, Shenyang, despite its advantages in population size, healthcare facilities, and economic development, shows poor resource matching because of its very large older adult(s) population. Through in-depth analysis of these contrasting cases, this study provides targeted recommendations and offers a geographical perspective for future research on older adult(s) care resources and regional disparities. Accordingly, the planning and construction of older adult(s) care facilities should be guided by a province-wide strategic perspective.
3. In terms of research regions, previous studies have largely focused on densely populated and economically developed areas (47), such as the Yangtze River Delta (49), Beijing (51), and Shandong Province (22). By selecting Liaoning Province in Northeast China as the study area, this research enriches the theoretical framework of related studies. Liaoning ranks first nationwide in terms of aging severity and has entered a stage of severe population aging, with a large and continuously growing older adult(s) population. Compared with populous central provinces such as Hunan, Liaoning faces weaker economic vitality and a more aged population structure, making the development of its older adult(s) care sector particularly urgent. Unlike highly developed regions such as the Yangtze River Delta, Northeast China is confronted with low fertility rates, population outflow, and increasing pressure on older adult(s) care services. As a former industrial base, Liaoning also faces challenges including economic decline, youth outmigration, and shortages of older adult(s) care resources. These compounded pressures make Liaoning a representative and globally relevant case. Studying its older adult(s) care resources not only helps improve the quality of life of the local older adult(s) population but also provides valuable experience for national strategies to address population aging.
4. With respect to research methodology, previous studies have mainly emphasized overall spatial distribution patterns, with relatively limited attention to the matching degree of older adult(s) care resources. This study introduces the consistency coefficient to classify the 14 prefecture-level cities in Liaoning Province into different resource-type categories and conducts in-depth analysis for each type. Among resource-surplus cities, those with surplus resources resulting from small population size, such as Benxi, are distinguished. For cities with relatively balanced resources, a further distinction is made between those achieving balance through high medical standards and favorable environmental conditions, such as Dalian, and those achieving balance despite weaker economic foundations due to smaller urban populations, such as Liaoyang. This refined classification allows for more precise and differentiated recommendations for older adult(s) care resource planning and development in Liaoning Province. Overall, classifying cities according to their older adult(s) care resource profiles constitutes a central contribution of this study. By comparing resource matching levels across different city types, it provides a scientific basis for addressing regional imbalances in older adult(s) care resources, improving resource allocation efficiency, and promoting the sustainable development of the older adult(s) care industry.
5. Regarding discussions on the planning of older adult(s) care facilities, Fan et al. argue that urban development should prioritize related infrastructure projects to further enhance accessibility and environmental sustainability, ensuring that urban growth benefits all residents, especially those in underdeveloped areas (53). Accessibility varies significantly across cities in Liaoning Province. At the provincial level, core cities such as Shenyang and Dalian have dense transportation networks in their old urban districts, allowing seniors to access older adult(s) care facilities relatively conveniently by bus, subway, or on foot. In contrast, areas with low to moderate accessibility feature sparse facility distribution and limited public transportation coverage, resulting in poor actual accessibility. At the regional level, the central–southern Liaoning urban cluster centered on Shenyang and Dalian exhibits high urban density and a well-developed road network, achieving overall accessibility levels far exceeding those of other regions in the province. Conversely, the mountainous areas of eastern Liaoning and the hilly regions of northwestern Liaoning have sparse populations and inadequate transportation infrastructure, leading to higher costs for seniors to access older adult(s) care services. Therefore, when considering accessibility, regional planning of older adult(s) care resources should not only focus on resource matching but also integrate factors such as transportation accessibility, urban infrastructure, and levels of economic development. Guided by the concept of the 15-min city (54), the development of older adult(s) care resources in Liaoning should aim to establish “15-min” service zones, with service radii controlled between 500 and 1,000 meters. However, based on the current spatial distribution of older adult(s) care resources, this target has not yet been achieved. In terms of supply–demand balance, Liaoning faces a serious spatial mismatch: high-quality older adult(s) care resources are overly concentrated in central cities, while rural areas with higher aging levels suffer from severe shortages. Therefore, future planning of older adult(s) care resources in Liaoning should draw on the experience of more developed southern cities such as Shenzhen (53), improve transportation accessibility to older adult(s) care facilities, prioritize addressing spatial mismatches, and strive to establish a “15-min” older adult(s) care service circle.
6. Regarding research limitations, on the one hand, this study does not incorporate policy-related factors, such as land supply, fiscal subsidies, and community infrastructure as influencing variables. Land supply can expand the overall scale of service facilities, fiscal subsidies can inject capital and momentum into older adult(s) care development, and community infrastructure policies can provide more convenient services for the older adult(s). Therefore, future research should incorporate policy factors when analyzing the spatial distribution of older adult(s) care resources. On the other hand, this study lacks in-depth analysis of areas with insufficient older adult(s) care resources and poor transportation accessibility. Future studies should place greater emphasis on the spatial dynamics and underlying causes of resource shortages in cities lagging in older adult(s) care development.
  This study has certain limitations in the scale analysis of influencing factors. Although the basic data originates from 3,187 POI points of older adult(s) care facilities, due to limitations in the availability of key influencing factors (such as urban GDP, traffic accessibility, etc.), the geographic detector analysis employed municipal-level aggregation units (n = 14). On the other hand, the planning approval and resource allocation of older adult(s) care facilities in China are primarily managed at the prefecture-level city scale, and adopting this research scale can directly align with policy practices. However, this aggregation process inevitably loses the micro-level information on the distribution of facilities within the city, such as the uneven allocation of resources between urban and suburban areas, as well as among different communities, which remains inadequately revealed. Future research could, where data permits, conduct analysis at the district, county, or even street level to explore the influencing mechanisms of the spatial distribution of older adult(s) care facilities at a more refined scale.
  In summary, this study investigates the spatial distribution of older adult(s) care facilities in Liaoning Province and its influencing factors, providing a reference for the planning and development of older adult(s) care facilities in other regions. It has positive implications for the planning, classification, and development of older adult(s) care resources in China. Overall, this paper integrates theory and practice to analyze the spatial distribution of older adult(s) care facilities in Liaoning Province and their driving factors. By focusing on regions with smaller populations and more severe aging, areas that have received limited attention in previous studies, this research enriches the existing literature and yields scientifically sound and reliable conclusions.

## 6 Conclusion and recommendations

### 6.1 Conclusion

This study utilized POI data from 3,187 older adult(s) care facilities across 14 prefecture-level cities in Liaoning Province as sample points. Web crawling technology was used to collect the data, which were then analyzed using ArcGIS software. Research methods including the nearest neighbor index, kernel density analysis, consistency coefficient, and geographic detector were applied to examine the spatial distribution characteristics and influencing factors of older adult(s) care facilities in Liaoning Province. The conclusions are as follows:

1. Spatial Distribution Characteristics of Elderly Care Facilities in Liaoning Province. The nearest neighbor index results indicate that older adult(s) care facilities in Liaoning Province exhibit significant clustering at both the provincial and municipal levels. Kernel density analysis reveals an uneven spatial distribution characterized by “overall dispersion with scattered clusters,” forming a high-density core centered on Shenyang, a Bohai Rim agglomeration belt, and secondary cores centered on Dandong and Fuxin. This finding highlights a clear center–periphery pattern in Liaoning’s older adult(s) care resources, with facilities concentrated in Shenyang and Dalian. Such a distribution risks neglecting areas with relatively underdeveloped older adult(s) care resources.
2. Spatial Matching Characteristics of Elderly Care Facilities and Senior Population in Liaoning Province. The consistency coefficient results show that Liaoning has six cities with advanced older adult(s) care facility resources, accounting for 42.86% of the total. These cities are mainly characterized by smaller older adult(s) populations and low-to-moderate levels of economic development. Four cities (28.57%) exhibit basic matching of older adult(s) care facilities, and are generally characterized by higher levels of economic development. Another four cities (28.57%) show lagging older adult(s) care resources; except for Shenyang, these cities have relatively underdeveloped economies and medium-sized older adult(s) populations. This finding provides theoretical support for government policies to allocate additional older adult(s) care resources to economically underdeveloped regions with poor transportation accessibility.
3. Factors influencing the distribution of older adult(s) care facilities in Liaoning Province. According to the factor detection results of the geographic detector, the most influential factor affecting the spatial distribution of older adult(s) care facilities in Liaoning Province is the size of the older adult(s) population. Other significant factors (*q*-value > 0.8) include total population size, urban GDP, and the number of healthcare facilities. Interaction analysis indicates that the spatial distribution of older adult(s) care facilities results from the combined effects of multiple factors. All factors show a two-factor enhancement effect, with particularly strong influences observed when the older adult(s) population interacts with total population size, urban GDP, and the number of healthcare facilities. These findings provide theoretical support for multi-factor analyses of the spatial distribution of older adult(s) care resources and offer a scientific basis for government authorities to plan older adult(s) care resources from multiple perspectives.

### 6.2 Recommendations for optimizing the allocation of older adult(s) care resources

Liaoning Province is among the earliest regions in China to enter a stage of deep aging. The construction and layout of older adult(s) care facilities, as well as the rational allocation of older adult(s) care resources, are crucial for achieving high-quality economic and social development. Based on the findings of this study, the following recommendations are proposed:

1. Optimize the layout of older adult(s) care facilities. First, in resource-advantaged cities, efforts should focus on improving the quality of older adult(s) care services. Cities such as Benxi, Dandong, and Tieling, although equipped with relatively abundant older adult(s) care facilities, have smaller older adult(s) populations. These cities should emphasize improving service quality and standards by introducing professional care teams and enhancing facility intelligence, thereby increasing their attractiveness and drawing older adult(s) residents from surrounding cities. Second, in cities with basically balanced resources, the number of facilities should be moderately increased in line with urban development plans to meet future demand from population aging. Third, in resource-lagging cities, governments should guide and encourage private capital and social participation to invest in older adult(s) care facilities, while simultaneously improving the utilization efficiency of existing resources.
2. The distribution of older adult(s) care resources in Liaoning Province is uneven, with facilities mainly concentrated in cities such as Shenyang and Dalian, forming a clear center–periphery pattern. Therefore, government policy should prioritize rural areas, urban fringe zones, and older residential communities—where supply–demand imbalances are most pronounced—in terms of land allocation and financial subsidies.
3. Liaoning Province faces a shortage of older adult(s) care resources with adequate transportation accessibility. Urban areas should therefore promote diversified older adult(s) care models, reduce service radii, and strive to establish “15-min” older adult(s) care service zones. First, community-based care should be encouraged through the establishment of community service centers that provide day care, rehabilitation, and cultural activities, enabling seniors to access services within their neighborhoods. Second, home-based care should be supported by expanding government-purchased services and volunteer programs to deliver in-home nursing and meal services, thereby improving care quality. Third, Liaoning should leverage its natural and regional advantages to develop travel-based older adult(s) care models, attracting seniors from other regions for summer retreats and leisure living to meet diverse older adult(s) care needs.
4. Liaoning Province faces relatively weak economic vitality and an aging population structure, making the development of its older adult(s) care industry an urgent necessity. The province should vigorously promote smart older adult(s) care platforms, using internet-based technologies to mitigate shortages in older adult(s) care resources and poor accessibility. This approach is particularly important for extending services to rural areas and older adult(s) individuals with limited mobility.

## Data Availability

The original contributions presented in the study are included in the article/supplementary material, further inquiries can be directed to the corresponding author/s.

## Footnotes

1
https://yanglao.mca.gov.cn/

2
https://mca.gjzwfw.gov.cn/
